# Proteomic Analysis of the Role of the Adenylyl Cyclase–cAMP Pathway in Red Blood Cell Mechanical Responses

**DOI:** 10.3390/cells11071250

**Published:** 2022-04-06

**Authors:** Elif Ugurel, Evrim Goksel, Neslihan Cilek, Elif Kaga, Ozlem Yalcin

**Affiliations:** 1 Research Center for Translational Medicine (KUTTAM), Koç University, Istanbul 34450, Turkey; eugurel@ku.edu.tr (E.U.); egoksel18@ku.edu.tr (E.G.); ncilek18@ku.edu.tr (N.C.); 2 School of Medicine, Koç University, Istanbul 34450, Turkey; 3 Graduate School of Health Sciences, Koç University, Istanbul 34450, Turkey; 4 Health Application and Research Center, University of Health Sciences, Afyonkarahisar 03100, Turkey; ekaga34@gmail.com

**Keywords:** red blood cell deformability, capillary transit, shear stress, phosphorylation, cytoskeletal proteins

## Abstract

Red blood cell (RBC) deformability is modulated by the phosphorylation status of the cytoskeletal proteins that regulate the interactions of integral transmembrane complexes. Proteomic studies have revealed that receptor-related signaling molecules and regulatory proteins involved in signaling cascades are present in RBCs. In this study, we investigated the roles of the cAMP signaling mechanism in modulating shear-induced RBC deformability and examined changes in the phosphorylation of the RBC proteome. We implemented the inhibitors of adenylyl cyclase (SQ22536), protein kinase A (H89), and phosphodiesterase (PDE) (pentoxifylline) to whole blood samples, applied 5 Pa shear stress (SS) for 300 s with a capillary tubing system, and evaluated RBC deformability using a LORRCA MaxSis. The inhibition of signaling molecules significantly deteriorated shear-induced RBC deformability (*p* < 0.05). Capillary SS slightly increased the phosphorylation of RBC cytoskeletal proteins. Tyrosine phosphorylation was significantly elevated by the modulation of the cAMP/PKA pathway (*p* < 0.05), while serine phosphorylation significantly decreased as a result of the inhibition of PDE (*p* < 0.05). AC is the core element of this signaling pathway, and PDE works as a negative feedback mechanism that could have potential roles in SS-induced RBC deformability. The cAMP/PKA pathway could regulate RBC deformability during capillary transit by triggering significant alterations in the phosphorylation state of RBCs.

## 1. Introduction

Red blood cells (RBCs) are exposed to various shear stresses in the circulatory system owing to the vascular wall and other blood components. RBCs respond to physiological shear stress by elongating themselves and improving their deformability, a prerequisite for entering and traversing narrow capillaries [1]. The maintenance of shear-induced improvement of deformability is essential in physiological conditions. However, this improvement in RBC deformability is disturbed in hematological diseases, such as sickle cell disease and hereditary hemoglobinopathies, or other pathological conditions, such as diabetes mellitus, diabetic nephropathy, or nondiabetic hypertension [2,3,4]. The main factors that determine RBC deformability are intracellular viscosity, surface area–volume interaction [5], the structure of the cytoskeleton [6,7], the ionic content of the cytoplasm that mediates RBC hydration state [8], and the metabolic processes that control redox state and ATP levels [9,10,11]. The structural properties of the cytoskeletal proteins and their interactions with each other or integral transmembrane complexes can determine the viscoelasticity of the RBC membrane, which is associated with cell deformability [12,13]. The shear stress sensing mechanisms and the accommodation of the cytoskeleton under shearing forces are critical parameters in the maintenance of shear-induced deformability. Shear stress can activate Piezo1 channels in the RBC membrane; in other words, Piezo1 channels can ‘sense’ shear stress and mediate calcium entry when they are chemically or mechanically activated [14,15]. Shear stress can also induce the release of microvesicles [16] and ATP from RBCs [17], and the latter is also associated with Piezo1 activation [15]. These mechanisms could all have roles in the regulation of shear-induced deformability.

Erythroid precursor cells expel their nuclei and organelles to enhance the oxygen-binding capacity of mature RBCs and increase cell deformability. Mature RBCs lack coding nucleic acids, such as deoxyribonucleic acid (DNA). Although current studies have revealed the existence of small ribonucleic acid (RNA) in RBCs, such as microRNAs and long non-coding RNAs, an active translation mechanism to mediate newly synthesized proteins is not present [18]. Therefore, RBC proteins are derived from precursor erythroid cells and persist during the entire lifetime of RBCs. On the other hand, cellular functions, including ion transport mechanisms, cell responses to oxygenated and deoxygenated conditions, maintenance of the cytoskeleton, and cell deformability, are all regulated by RBC proteins. Given that the synthesis of novel proteins does not occur, the vital cellular functions would be maintained by post-translational modifications of the existing proteins via phosphorylation. Phosphorylation contributes to the pathogenesis of sickle cell disease [19,20,21] or malaria [22,23] and regulates redox stress [24] or the interactions between cytoskeletal and membrane proteins [25,26,27].

Previous studies reported that the adenylyl cyclase (AC)/protein kinase A (PKA) signaling pathway could be a modulator of RBC deformability [28,29,30]. We previously showed that this signaling pathway could have roles in maintaining shear-induced deformability in patients with sickle cell disease [31]. The activation of membrane-bound AC via G protein-coupled receptors (GPCRs) triggers the AC/PKA signaling pathway, which follows the conversion of ATP into cAMP. The elevation of intracellular cAMP levels activates PKA (cAMP-dependent protein kinase), which phosphorylates its targets, including RBC membrane proteins [32,33]. Protein phosphorylation via the AC/PKA pathway could alter cytoskeletal interactions with the RBC membrane and modulate RBC deformability. According to a recent study, the RBC phosphoproteome is modulated in response to deformation [34]. Therefore, the primary objective of this study was to examine the changes in the phosphorylation of tyrosine and serine residues of the proteins in the RBC proteome. We hypothesized that the phosphorylation status of membrane proteins would determine the shear-induced mechanical responses of RBCs. Our main objective was to examine the relationship between RBC deformability and the AC/PKA pathway and how they mediate the phosphorylation state of the RBC proteome. We also hypothesized that AC/PKA signaling mechanisms could modulate shear-induced RBC deformability through changes in the phosphorylation status of cytoskeletal proteins.

## 2. Materials and Methods

### 2.1. Blood Sampling and Drug Treatments

Peripheral blood samples from antecubital veins of 12 healthy individuals aged between 20–45 years were collected in K_3_EDTA (1.8 mg/mL) vacutainers. Informed consent was obtained from each donor. The study was approved by the Koç University Ethics Committee (IRB: 020/2012) and was performed in accordance with the declaration of Helsinki (The Code of Ethics of the World Medical Association). Complete blood count was assessed by an automated hematology analyzer (ABX Micros, ABX Diagnostics Inc, ABX Micros, Horiba ABX, Kyoto, Japan). The hematocrit level of blood samples was measured by capillary tubes using a microcentrifuge and adjusted to 0.4 L/L or 0.2 L/L with autologous plasma, depending on the experimental setup. An adenylyl cyclase inhibitor (SQ22536), a phosphodiesterase inhibitor (pentoxifylline), a protein kinase A inhibitor (H-89 dihydrochloride hydrate), and a Piezo1 inhibitor (gadolinium) were purchased from Sigma-Aldrich (St. Louis, MO, USA) and prepared in phosphate-buffered saline (PBS, pH 7.4; GIBCO, Invitrogen, Carlsbad, CA, USA) according to the recommendations of the supplier. Whole blood samples were incubated with SQ22536 (100 µM), H-89 (10 µM), pentoxifylline (10 µM) or gadolinium (30 µM) for 15 min at 37 °C. Sham-treated samples were studied as controls. Preparation of blood samples, incubation with the drugs, and deformability measurements were carried out within 4 h.

### 2.2. Application of Physiological Shear Stress to Blood

Two different experimental setups were followed to apply shear stress to the blood samples. In the first setup, a laser-assisted optical cell analyzer (LORRCA MaxSis, Mechatronics, The Netherlands) was used to generate 5 Pascal (Pa) shearing force which corresponds to a physiological shear stress level at arterial walls [35]. The LORRCA device has a Couette type shearing system with inner (static) and outer (rotating) cylinders. The blood sample is diluted with an isotonic medium (PVP (polyvinylpyrrolidone) ISO osmolar, pH 7.4, Mechatronics, The Netherlands) to a 1:200 ratio, and the suspension is applied into the measuring chamber, the space between the two cylinders. The shearing force is driven by the rotating cylinder and the viscosity of the suspending medium (PVP, viscosity: 29.8 mPa.s). The hematocrit level of blood was adjusted to 0.4 L/L before the dilution. The shearing process was performed at 37 °C for 300 s to reach a significant level of RBC mechanical response [36].

For the second experimental setup, a cylindrical capillary tubing system (1 m in length and 0.05 cm in diameter) was connected to a syringe pump (KD Scientific, Legato 210, Holliston, MA, USA) and placed in a water bath at 37 °C. First, blood viscosity was measured by a cone-plate viscometer (Programmable DV-II + Viscometer, Brookfield). Then, the flow rate (mL/min) in the capillary tubing system was calculated by the geometry of the capillary tube and the blood viscosity according to the following formula (Poiseuille’s law) to yield 5 Pa SS:

∆*p* = 8μLQ/(πR^4^) (1)

where Δ*p* is the pressure difference between the two ends of the tube (5 Pa), L is the length of the tube, μ is the dynamic viscosity, Q is the volumetric flow rate, and R is the tube radius [37,38].

The hematocrit level of blood was adjusted to 0.2 L/L with autologous plasma and transfused slowly into the system with a syringe (Becton Dickenson, Franklin Lakes, NJ, USA) connected to the pump and the tubing. The system was airtight except for the open end of the tube where the sample was collected. The syringe pump was adjusted to the calculated flow rate with a target volume of 2 mL. The shear stress was applied by back-and-forth flowing for 300 s.

### 2.3. RBC Deformability Measurement

RBC deformability was evaluated by the LORRCA MaxSis. The device has a diode laser integrated with the static cylinder, and the laser beam is diffracted by RBCs as it traverses through the blood sample. The diffraction pattern is monitored by a CCD camera and analyzed by an integrated computer.

RBC deformability is evaluated as it is elongated in horizontal and vertical directions under a shear stress range (0.3–50 Pa) [39]. The measurements of RBC deformability are recorded as elongation index (EI), which is calculated as follows: EI = (a − b)/(a + b), where “a” is the vertical axis and “b” is the horizontal axis of the diffraction pattern. Deformability curves were obtained by plotting EI versus applied SS level. Maximal RBC elongation index (EImax) and the SS required for one-half of this maximal deformation (SS1/2) were calculated by the linear Lineweaver–Burke model [40]. The SS1/2 parameter provides an index to RBC deformability, while EImax indicates the limiting elongation index at infinite shear stress [40]. The SS1/2:EImax ratio was calculated as a normalized measure of SS1/2 [41]. Deformability measurements were taken before and after applying physiological shear stress (5 Pa) to blood for 300 s. All of the experiments were performed at 37 °C.

### 2.4. RBC Membrane Isolation and Protein Preparation

RBCs were isolated from whole blood by density gradient separation to eliminate platelet-containing plasma, granulocytes, lymphocytes, and other mononuclear cells [42]. Accordingly, Histopaque-1119 (Sigma-Aldrich) and Histopaque-1071 (Sigma-Aldrich) solutions were layered on top of each other in a 15 mL canonical tube, and the blood was layered on top of the solutions. RBCs were collected at the bottom of the tube after centrifuging at 700× *g* for 30 min at room temperature and washed with isotonic phosphate-buffered saline solution (150 mm NaCl, 5 mm sodium phosphate dibasic, 1 mm PMSF, 1mm sodium orthovanadate, pH 7.4). RBC membranes (white ghosts) were prepared according to the method of Pesciotta et al. with the following modifications [43]. First, RBCs were lysed in 10 volumes of hypotonic lysis buffer (5 mm sodium phosphate dibasic, 1 mm EDTA, 1 mm PMSF, 1mm sodium orthovanadate, pH 8) for 15 min at +4 °C. Then, the suspensions were centrifuged at 40,000× *g* for 15 min at +4 °C using a total of six washes until the ghosts became whitish and the supernatant appeared colorless. Next, RBC membranes were solubilized in rehydration sample buffer (BioRad, Hercules, CA, USA) containing 8 M urea, 2% CHAPS, 50 mm DTT, 0.2% Bio-Lyte^®^ 3/10 ampholyte. Proteins were extracted with vigorous shaking for 30 min at room temperature. The samples were centrifuged at 10,000× *g* for 15 min, and supernatants were collected. Protease inhibitor cocktail (Roche Applied Science, Penzberg, Germany) and phosphatase inhibitor cocktail (Roche Applied Science, Germany) were added according to the manufacturer’s recommendations, and the protein aliquots were stored at −80 °C.

### 2.5. Proteomics Experimental Design

Proteomics studies were designed to include five treatment groups: SQ22536, H-89 dihydrochloride hydrate, and pentoxifylline treated blood samples exposed to 5 Pa SS and two untreated blood samples of which only one was exposed to 5 Pa SS by the capillary tubing system. Blood samples were collected from three healthy donors and studied with three biological replicates. Protein concentration was assessed by the Bradford method using a Quick Start Bradford Protein Assay (BioRad, CA, USA). Each sample (100 µg protein) was applied onto immobilized pH gradient strips (ReadyStrip IPG strips, 7 cm, pH 4–7, BioRad, CA, USA) and rehydrated for 12 h with active rehydration at 50 V in a Protean IEF Cell (BioRad). Isoelectric focusing was performed at 20 °C with the following conditions: increasing the voltage at 250 V for 20 min, ramping to 4000 V for 2 h, and then focusing to reach 20,000 Vh. The strips were equilibrated for 15 min in the equilibration buffer containing 6 M urea, 2% SDS, 0.375 M Tris-HCl (pH 8.8), 20% glycerol, 2% DTT, and equilibrated for another 15 min in the equilibration buffer with the replacement of DTT with 2.5% iodoacetamide. The strips were embedded in 0.5% overlay agarose (BioRad) and placed on precast acrylamide gels (Any kD Mini-PROTEAN TGX Stain-Free Protein Gels, BioRad). Electrophoresis was performed at 200 V with Tris–glycine–SDS buffer (25 mm Tris, 192 mm glycine, 0.1% SDS, pH 8.3, BioRad). The gels were stained with SYPRO Ruby (BioRad) and scanned with the Gel Doc XR+ System (BioRad). Protein spot analysis from scanned gels was performed using PDQuest software version 7.1 (BioRad). The untreated sample before shear stress application was selected as a reference gel. Each spot’s relative volume and intensity were calculated and normalized to eliminate the differences due to sample loading and gel staining. Spots with more than a 1.5-fold difference in volume and intensity were selected for LC-MS/MS analysis. Protein spots were further stained with Coomassie blue and excised from the gel with sterile pipette tips.

### 2.6. In Gel Digestion, nLC-MS/MS, and Data Acquisition

Excised gels were destained in 150 µL of 200 mm ammonium bicarbonate/50% acetonitrile for 30–60 min and vacuum dried. Gels were then incubated with 10 μL of 25 ng/μL trypsin solution prepared in 200 mm ammonium bicarbonate and incubated at 37 °C for 14 h. Peptide extraction was carried out in 100 µL of 60% acetonitrile/0.1% trifluoroacetic acid solution by incubation at 30 °C for 40 min, and supernatants were collected after centrifugation. This step was repeated twice, then supernatants were pooled and vacuum dried in a concentrator (Speed Vac concentrator, Thermo Scientific, Waltham, MA, USA). Peptide samples were dissolved in 5 µL of 5% acetonitrile/5% trifluoroacetic acid solution and enriched in the C18 column (stage type). Peptides were separated in a nano-LC column (C18, 3 µm, Gold200, Dr. Masch) with a mobile phase of 95% of solvent A (5% acetonitrile/0.2% formic acid) and 5% of solvent B (99.8% acetonitrile/0.2% formic acid) using a flow rate of 300 nL/min. Samples were analyzed in a Q Exactive Quadrupole-Orbitrap Mass Spectrometer (Thermo Scientific, USA) for peptide identification by Thermo Discoverer 1.4 software (Thermo Scientific, USA) at a resolution of 70,000 (*m*/*z*). Analysis was performed in replicates. A data search was carried out by Mascot (Version 2.4, Matrix Science, London, UK) and SEQUEST data servers against the UniProt knowledgebase database (UniProtKB). Peptides with high confidence (0.01% false discovery rate) were selected for the identification.

### 2.7. Validation Experiments

RBC protein samples were prepared according to the Laemmli protocol [44]. Briefly, 30 µg protein from each treatment group was diluted with 4X Laemmli buffer (BioRad) and reduced with 2-mercaptoethanol (1:10). The samples were boiled at 95 °C for 5 min and loaded onto 4–10% SDS–polyacrylamide gels. Electrophoresis was performed by applying a voltage gradient: 100 V for 15 min, 150 V for 15 min, 180 V for 10 min, and 200 V for 10 min. Proteins were transferred onto PVDF membranes (BioRad) in the Trans-Blot Turbo Blotting system (BioRad) by applying 1.3 A up to 25 V for 10 min. PVDF membranes were incubated in a blocking buffer containing 20 mM Tris, 150 mm NaCl, 0.1% Tween-20, and 5% BSA (Bovine serum albumin, Sigma, Sigma-Aldrich, St. Louis, MO, USA) for 1 h. Then, PVDF membranes were incubated at 4 °C overnight with primary antibodies for alpha-spectrin (Abcam, Cambridge, UK), dematin (Abcam), tropomodulin (Abcam), tropomyosin (Cell Signaling, Danvers, MA, USA), protein 4.1 (Abcam), or actin (Abcam), in addition to GAPDH (Cell Signaling) as a housekeeping protein in each assay. PVDF membranes were washed with TBS-T solution (20 mm Tris, 150 mm NaCl, and 0.1% Tween-20), and incubated for 1 h in secondary antibody solution containing anti-rabbit (Abcam, 1:5000) or anti-mouse (Abcam, 1:5000) antibodies. PVDF membranes were washed again with TBS-T solution and incubated in blotting substrate (ECL Western Blotting Substrate, Pierce) according to the manufacturer’s recommendations. Protein bands were scanned in the chemiluminescent channel in an imaging system (LI-COR Odyssey Fc, Lincoln, NE, USA). The protein intensities were calculated by Image Studio software (LI-COR, US) and normalized according to GAPDH intensity. The studies were performed in replicates.

### 2.8. Protein Phosphorylation Studies

Protein phosphorylation studies were performed by Western blot and immunofluorescence methods. For the Western blot, 30 µg protein from each treatment group was dissolved in Laemmli buffer (BioRad) containing 355 mm 2-mercaptoethanol and incubated at 95 °C for 5 min. Protein samples were loaded onto 4–10% polyacrylamide gels and separated by applying a voltage gradient: 100 V for 15 min, 150 V for 15 min, 180 V for 10 min, and 200 V for 10 min. Gels were either stained with SYPRO Ruby or proceeded with the Western blot. Accordingly, proteins were transferred onto PVDF membranes (BioRad) in the Trans-Blot Turbo Blotting system (BioRad) by applying 1.3 A up to 25 V for 10 min. PVDF membranes were incubated in a blocking buffer containing 20 mm Tris, 150 mm NaCl, 0.1% Tween-20, and 5% BSA (Bovine serum albumin, Sigma) for 1 h. Anti-phosphotyrosine (Sigma) and anti-phosphoserine (Sigma) antibodies were prepared in 1:1000 dilution in the blocking buffer. PVDF membranes were incubated in antibody solutions at 4 °C for 14–16 h. After washing with TBS solution (20 mm Tris, 150 mm NaCl) with 0.1% Tween-20, membranes were incubated for 1 h in secondary antibody solution containing anti-rabbit (Abcam, 1:5000) or anti-mouse (Abcam, 1:5000) antibodies prepared in blocking buffer. Membranes were washed with TBS solution with 0.1% Tween-20 twice and incubated in blotting substrate (ECL Western Blotting Substrate, Pierce) according to the manufacturer’s recommendations. Proteins on PVDF membranes were scanned in the chemiluminescent channel in an imaging system (LI-COR Odyssey Fc, US). Image analysis was performed using Image Studio software (LI-COR, US). Phosphorylated protein bands were identified on stained gels and excised with a sterile scalpel for LC-MS/MS analysis.

Immunofluorescence was performed according to Pan et al. with minor modifications [45]. Briefly, glass coverslips (dia. 12 mm) were acid-washed and coated with 0.1 mg/mL poly-L-lysine for 3 h at room temperature. Then, 4 µL of fresh human blood was diluted in 6 mL phosphate-buffered saline (PBS) containing 10 mM glucose and 5 mg/mL BSA (PBS-GB) and washed once with PBS-GB by centrifugation at 1200 rpm for 5 min. RBCs were suspended in a fixation solution of 3% paraformaldehyde and 0.1% glutaraldehyde in PBS for 10 min, centrifuged at 1200 rpm, and resuspended in PBS at ~3 × 10^6^ cells/mL. The fixed RBCs were allowed to adhere to the poly-L-lysine coated coverslips for ~4 h and then permeabilized with 0.05% Triton X-100 in PBS for 1 h. RBCs were stained with either phosphotyrosine recombinant rabbit monoclonal antibody (Invitrogen, JA10-49, 1:50) or anti-phosphoserine mouse monoclonal IgG1 (Millipore, clone 4A4, 1:100) overnight. Coverslips were washed once with PBS-GB and incubated with secondary antibodies (Alexa Fluor 488 anti-rabbit or Alexa Fluor 488 anti-mouse, Ex/Em: 490/525 nm, 1:1000) in 3% BSA in PBS for 1 h. Stained RBCs were detected at the 488 nm channel for phospho-staining and the 532 nm channel for RBC autofluorescence using a Leica DMI8 SP8 inverted Confocal Microscope with a 63X magnification. Data were collected from 5 different areas of each slide (approximately 200 cells per slide) and studied in triplicates. We investigated the images in LAS X software (Leica, Wetzlar, Germany) and used the zoom factor tool for the presentation.

### 2.9. Statistical Analysis

Results were recorded as means ± SD unless otherwise stated. All statistical analyses were performed with GraphPad Prism 7.0 (GraphPad Software Inc., La Jolla, CA, USA). The significance level was defined as *p* < 0.05. Deformability data were analyzed by two-way ANOVA, followed by Bonferonni’s multiple comparisons test. Western blot data were analyzed by one-way ANOVA, followed by Dunnett’s multiple comparisons test. Deformability experiments were studied in replicates. All proteomics and phosphorylation studies were performed in triplicates.

## 3. Results

### 3.1. cAMP/PKA Signaling Pathway and Piezo1 Contribute to Shear-Induced RBC Deformability

**Figure 1:**
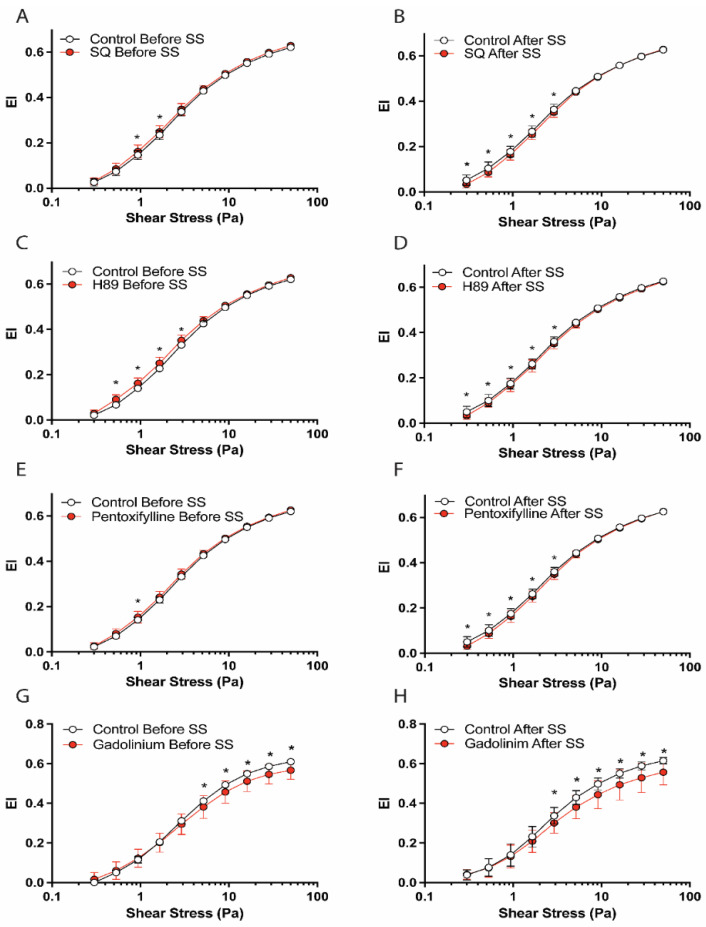
EI–SS curves before and after 5 Pa SS exposure. The graphs demonstrate deformability changes by SQ22536 (**A**,**B**), H89 (**C**,**D**), pentoxifylline (**E**,**F**), and gadolinium (**G**,**H**) compared to the control. Panels (**A**,**C**,**E**,**G)** show deformability curves before SS exposure and panels (**B**,**D**,**F**,**H)** show deformability curves after SS exposure. Data are represented as means ± SD, n = 12, * *p* < 0.05.

Intracellular signaling is initiated by the activation of membrane-bound adenylyl cyclase (AC), which converts ATP to cAMP. The intracellular signal is transmitted via cAMP-binding proteins or cAMP-dependent protein kinase (PKA) by phosphorylating the target proteins. Therefore, we investigated the roles of adenylyl cyclase on the regulation of shear-induced RBC deformability. The inhibition of AC by SQ22536 improved RBC deformability before shear stress inducement (Figure 1A). Interestingly, the implementation of SQ22536 significantly deteriorated RBC deformability under shear stress (SS) (Figure 1B). SS1/2 and EImax parameters are shown in Supplementary Figure S1. Accordingly, the normalized ratio of EImax (SS1/2:EImax) was significantly increased by SQ22536 after SS exposure, which indicates an impairment in deformability. Therefore, adenylyl cyclase may have a regulatory role in RBC deformability, which is strongly modulated by the effect of shear stress. In physiological conditions, the activation of adenylyl cyclase leads to an increase in intracellular cAMP concentration, which can activate protein kinase A (PKA) or cAMP-binding proteins (e.g., Epac). We used a selective inhibitor of PKA, H89 dihydrochloride, that significantly improved RBC deformability before shear exposure. However, RBC deformability after shear exposure was substantially impaired by H89 dihydrochloride treatment (Figure 1C,D).

**Figure 2:**
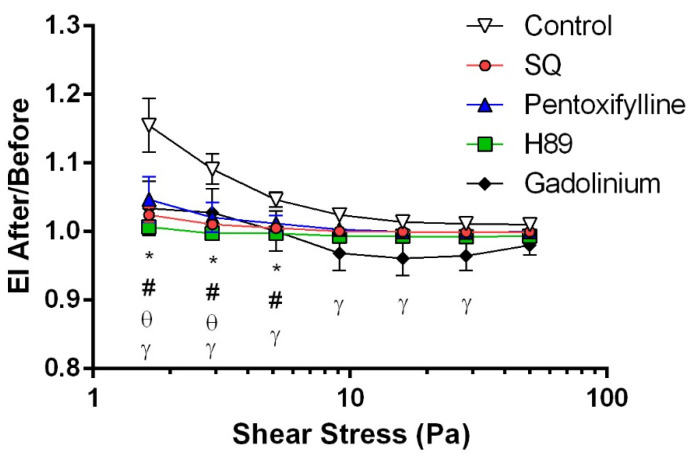
Ratio of the elongation index (EI) values measured after and before the application of 5 Pa shear stress. * *p* < 0.05 control vs. SQ, θ *p* < 0.05 control vs. pentoxifylline, # *p* < 0.05 control vs. H89, and γ *p* < 0.05 control vs. gadolinium. Data under 0.95 Pa are not plotted. Data are represented as means ± SE, n = 12.

The intracellular signal mediated by cAMP is attenuated by phosphodiesterase (PDE), which converts cAMP to AMP. To investigate the effects of various PDE enzymes on RBC deformability, we implemented pentoxifylline, a non-selective PDE inhibitor, which significantly improved RBC deformability before the shearing procedure but impaired it after shear stress application (Figure 1E,F). On the other hand, shear stress could activate the Piezo1 channel on the RBC membrane. To examine the role of Piezo1 on shear-induced deformability, gadolinium, a Piezo1 inhibitor, was implemented for blood samples. Accordingly, gadolinium significantly deteriorated RBC deformability before and after shear stress exposure (Figure 1G,H). The impairment in deformability was recorded from moderate (5.15 Pa) to high (50 Pa) shear stress levels. In addition, we calculated EI after/before ratio over the range of shear stress levels, as previously described [46]. Accordingly, EI after/before ratios of SQ22536 and pentoxifylline are significantly lower than the control group at 1.65 and 2.91 Pa levels (*p* < 0.05, Figure 2). EI after/before ratios of H89 and gadolinium are significantly different from the control in a shear stress range of 1.65–5.15 Pa and 1.65–28.32 Pa (*p* < 0.05, Figure 2).

### 3.2. Shear Stress and cAMP/PKA Signaling Do Not Alter Protein Composition in RBC Membranes

**Figure 3:**
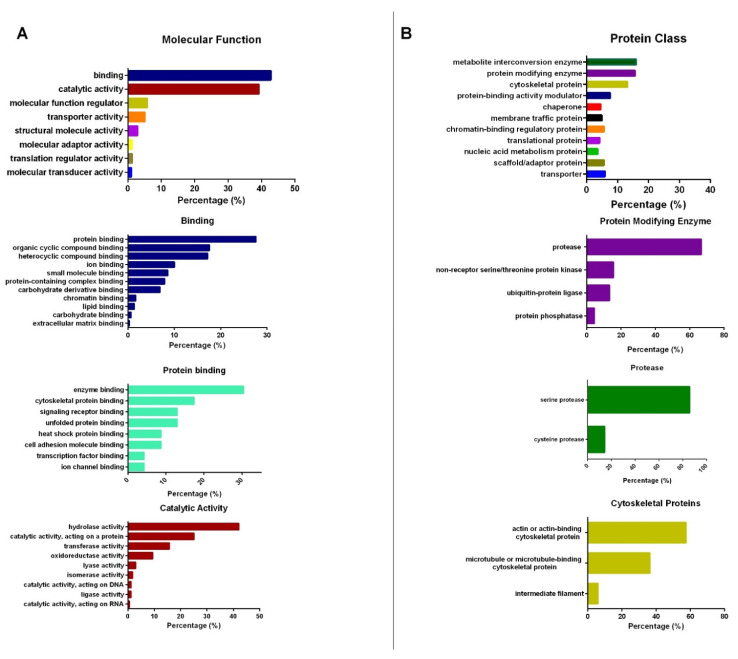
The categorization of proteins according to their molecular function (**A**) and protein class (**B**). The majority of the proteins are protein-modifying enzymes and cytoskeletal proteins (**B**) and have functions of binding and catalytic activity (**A**).

Common protein spots (n = 116) were identified and analyzed according to their intensity. Spots (n = 15) with more than 1.5-fold difference in volume and intensity were selected and analyzed by LC-MS/MS. Identified proteins were further analyzed by the PANTHER classification system (www.pantherdb.org accessed on 29 December 2021) and depicted as bar plots. Proteins were categorized according to their molecular function and protein class. The majority of the proteins have binding or catalytic activity (Figure 3A). The binding activity is mainly related to protein binding, organic cyclic compound binding, and heterocyclic compound binding, while the catalytic activity of the proteins is mainly related to hydrolase activity and catalytic activity acting on a protein. The binding function of the proteins mostly includes enzyme binding (e.g., GTPase binding), cytoskeletal protein binding (e.g., actin binding), and signaling receptor binding (e.g., G protein coupled receptor binding). When proteins were categorized according to their protein class, most of the proteins belonged to metabolite interconversion enzymes, protein-modifying enzymes, and cytoskeletal protein classes (Figure 3B). Protein-modifying enzymes include proteases and serine/threonine-protein kinase, while cytoskeletal proteins include actin/actin-binding and microtubule/microtubule-binding cytoskeletal proteins. Protease activity is related to serine and cysteine proteases. All proteins were identified according to their coverage, unique peptide number, and peptide sequence match; they are listed in Table 1. More than one protein spot might refer to the same protein. Proteins whose molecular function and protein class were related to protein binding and the cytoskeleton were further selected for validation experiments. Accordingly, we selected actin, protein 4.1, spectrin alpha, tropomyosin alpha-1, tropomodulin-1, and dematin to validate their differential expressions. Supplementary Figure S2 shows protein expression levels in five treatment groups. Although there were slight variations among the study groups, no significant difference was recorded (Supplementary Figure S2). Therefore, the exposure of shear stress and the modulation of the cAMP/PKA signaling pathway did not significantly alter protein expression or composition in the RBC membrane.

### 3.3. Shear Stress and cAMP/PKA Signaling Induce Changes in Phosphorylation

**Figure 4:**
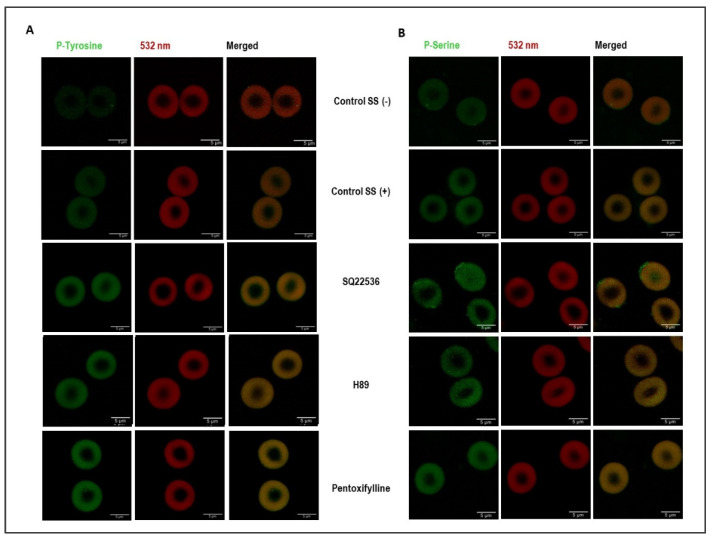
The changes in the phosphorylation status of RBCs due to different treatments. Tyrosine and serine phosphorylation are shown in green (488 nm) in panels (**A**,**B**), respectively.

The shear stress exposure induced a significant increase in both serine and tyrosine phosphorylation of the intact cells (Figure 4). PKA inhibition by H89 dihydrochloride increased tyrosine phosphorylation relative to the controls; however, serine phosphorylation was approximately the same as the sheared control. The inhibition of adenylyl cyclase by SQ22536 induced phosphorylation in both serine and tyrosine residues compared to the controls. The inhibition of PDE by pentoxifylline substantially increased tyrosine phosphorylation compared to the controls (Figure 4A). However, the pentoxifylline treatment did not change serine phosphorylation compared to sheared control (SS+) (Figure 4B). Phosphorylation status in intact RBCs by different drug treatments before 5 Pa SS exposure is shown in Supplementary Figure S3.

**Figure 5:**
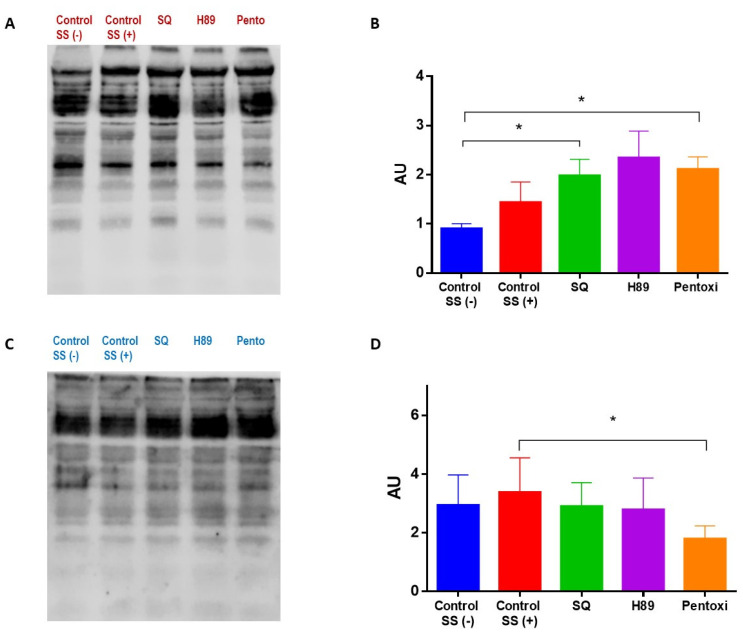
The changes in the phosphorylation of RBC membrane proteins due to different treatments. Phosphorylation at tyrosine and serine residues of cytoskeletal proteins are depicted in panels (**A**,**B**) and (**C**,**D**), respectively. Panels (**A**,**C**) are representative images. Data are represented as means ± SD, n = 10, * *p* < 0.05.

Physiological shear stress slightly increased both tyrosine and serine phosphorylation in RBC membrane proteins (Figure 5). Phosphorylated protein bands were also excised and analyzed in an independent run by LC-MS/MS. We identified spectrin, band 3, protein 4.1, protein 4.2, 55 kD erythrocyte membrane protein, dematin and tropomodulin to be phosphorylated. We evaluated the changes in serine and tyrosine phosphorylation in RBC membrane proteomes due to different treatments. Accordingly, the inhibition of adenylyl cyclase by SQ22536 significantly increased tyrosine phosphorylation (*p* < 0.05) but did not alter serine phosphorylation. The inhibition of PKA by H89 dihydrochloride increased tyrosine phosphorylation but did not significantly change serine phosphorylation of membrane proteins. The inhibition of PDE by pentoxifylline significantly increased tyrosine phosphorylation compared to the unsheared control (SS−) (*p* < 0.05, Figure 5A). On the other hand, serine phosphorylation was significantly decreased by pentoxifylline compared to the sheared control (SS+) (*p* < 0.05, Figure 5B).

## 4. Discussion

In the present study, we have demonstrated that cAMP-dependent signaling could regulate RBC deformability during capillary transit (1), that the cAMP/PKA pathway primarily targets protein-modifying enzymes and cytoskeletal proteins in RBCs (2), that the modulation of the cAMP/PKA pathway alters the phosphorylation of RBC proteins without changing protein abundance in the membrane (3), and that phosphorylation levels may determine the extent of RBC deformability (4). We also demonstrate that physiological shear stress in capillaries improves RBC deformability by inducing mild phosphorylation in the RBC proteome (5). These results are consistent with the previous findings [34] that the activity of protein kinases is required to improve RBC deformability as a response to shear stress. However, the cAMP/PKA pathway modulation under shearing conditions impairs RBC deformability by inducing a significant increase in the phosphorylation status of RBCs (6). Therefore, in this study, we report for the first time that shear-induced deformability, which is essential for RBCs to pass through narrow capillaries, could be regulated by the level of the phosphorylation of cytoskeletal proteins, possibly through the cAMP/PKA pathway which determines protein–protein interactions within the cell membrane and the mechanical stress responses of RBCs.

**Figure 6:**
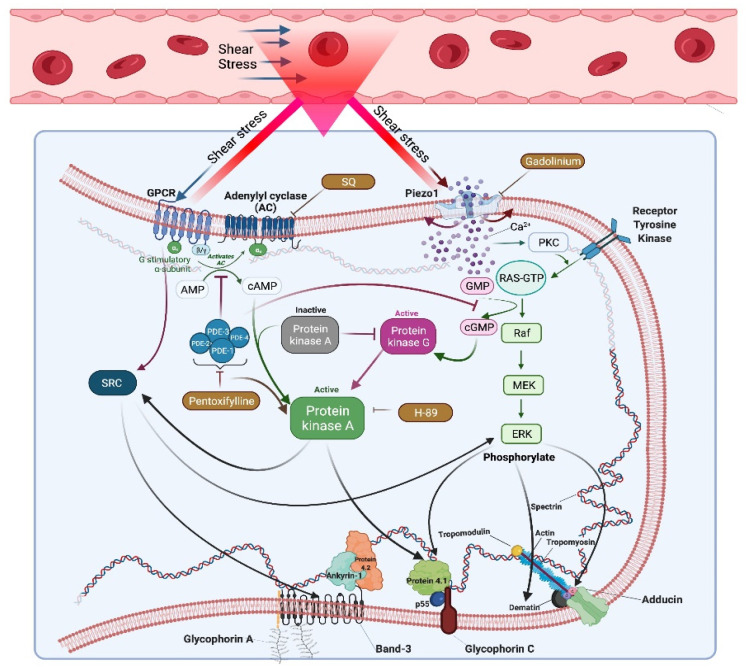
A schematic illustration for the phosphorylation events through cAMP signaling mechanisms. Shear stress in blood flow could be sensed via Piezo1 and GPCRs that trigger cAMP signaling or crosstalk between signaling pathways. Closed lines show an inhibition and black arrows represent a phosphorylation event. GPCR: G protein-coupled receptors, PDE: Phosphodiesterase, PKC: Protein kinase C.

Various studies have suggested that RBC deformability could be affected and regulated by intracellular signaling mechanisms, including the cAMP/PKA pathway [30,47,48], Ca^2+^ signaling mechanisms [28,49], the Rho/Rho kinase pathway [50,51], and the nitric oxide synthase signaling pathway [52,53,54]. However, the role of signaling mechanisms in RBC mechanical stress responses and the relevant changes in the RBC proteome had not been investigated. We previously showed that the cAMP/PKA pathway could potentially be a therapeutic approach for treating sickle cell disease due to shear-induced improvements of RBC deformability [31]. In the present study, we focused on the cAMP/PKA pathway regarding its possible effects on the RBC proteome and post-translational modifications of membrane proteins associated with the cytoskeletal structure that could determine shear-induced deformability in healthy RBCs. We investigated deformability changes before and after shear stress exposure with a phosphodiesterase (PDE) inhibitor (pentoxifylline), a protein kinase A (PKA) inhibitor (H89 dihydrochloride), and an adenylyl cyclase (AC) inhibitor (SQ22536). Accordingly, these inhibitors increased RBC deformability before shear stress exposure; however, they significantly impaired shear-induced deformability (after shear stress). These results require considerable attention since shear stress at the physiological level improves RBC deformability and elongates and orients the cells into the shear flow [55,56]. From this perspective, the expected increase in the elongation index of RBCs under shearing conditions is impaired by the inhibition of the enzymes in the cAMP/PKA pathway. Therefore, AC, PKA, and PDE, the critical components of this pathway, could have important roles in RBC deformability during capillary transit. We further investigated the role of the Piezo1 channel on shear-induced RBC deformability by implementing gadolinium, a nonspecific channel inhibitor. Piezo1 is activated by shear stress or membrane stretching and acts as a mechanosensor in RBCs. The mechanotransduction mediated by Piezo1 leads to Ca^2+^ influx and contributes to cell volume homeostasis. The inhibition of Piezo1, in the present study, significantly impaired RBC deformability at moderate-to-high shear stress levels compared to the control, although deformability at low shear stress regions was not significantly changed. These results are consistent with a previous report that RBC deformability is not affected by the inhibition of Piezo1 at lower stress levels (~3.5–4 Pa) [15]. However, we showed that RBC deformability was significantly impaired from moderate-to-high stress levels (5.15–50 Pa) by Piezo1 inhibition. Therefore, we suggest that the function of the Piezo1 channel is essential for the maintenance of RBC deformability in narrow capillaries where shear stress levels are high. The function of Piezo1 would also be necessary for RBCs circulating in extracorporeal assist devices or artificial organs, which implement higher SS levels than normal physiological conditions. Enabling Ca^2+^ influx by the activation of Piezo1 may lead to the triggering Ca^2+^-dependent mechanisms, including the protein kinase C (PKC) signaling pathway. We did not rule out the effects of this mechanosensitive channel on shear-induced deformability, but the associations of Piezo1 with intracellular signaling mechanisms have yet to be determined. Previously, we demonstrated that the inhibition of PKC ameliorated calcium-impaired deformability under conditions of continuous shear, although this inhibition alone did not significantly affect shear-induced deformability [57]. We seriously consider that the contribution of PKA signaling mechanisms to shear-induced deformability is more significant than that of PKC signaling based on the findings of the present study. This would support the phenomenon of a possible ‘’crosstalk’’ between intracellular signaling pathways to regulate RBC deformability under continuous shear stress (Figure 6).

Mass spectrometry analysis in the present study revealed that protein-modifying enzymes, including serine/threonine protein kinases and proteases, and cytoskeletal proteins, such as actin/actin-binding cytoskeletal proteins, could be responsible for the maintenance of shear-induced deformability. The exposure of shear stress or cAMP-dependent cellular signals conceivably would have affected protein-modifying enzymes; however, they can also change or activate actin-binding cytoskeletal proteins. Therefore, we focused more on this protein class since the structure of the cytoskeletal proteins could determine RBC deformability. We found no significant change in the expression of actin, spectrin, protein 4.1, tropomodulin, tropomyosin, and dematin by the implementation of shear stress and the inhibition of AC, PKA, and PDE enzymes. These data are not surprising, since RBCs lack nuclei or nucleic acids to express novel proteins in demand. However, we did not exclude that cytoskeletal proteins might undergo a degradation process due to shear stress exposure or cAMP-driven cellular signals because mass spectrometry results showed that the proteases could be differentially expressed along with the serine/threonine protein kinases. In addition, shear stress-triggered Piezo1 activation enables calcium entry and activates calpains, Ca^2+^-dependent proteases, which facilitate protein cleavage and degradation [58,59]. However, we found no significant change in the expression of cytoskeletal proteins, which shows that the activation of proteases or the inhibition of protein kinases did not substantially affect the integrity of the RBC membrane proteome.

In the present study, shear stress exposure did not alter the RBC proteome but induced changes in the phosphorylation status of RBC proteins in both the membrane and the intact cell. Shear-induced phosphorylation might be mediated by Piezo1 and G protein-coupled receptors (GPCRs) such that the latter directly interacts with Src-family kinases, phosphorylating tyrosine residues of target proteins [60,61,62]. Previously, Band 3, an integral transmembrane protein that links the RBC membrane to the cytoskeleton, was shown to be phosphorylated by Syk and Lyn, which are members of the Src family [63,64]. A recent study demonstrated that the inhibition of Syk tyrosine kinase by imatinib blocked band 3 tyrosine phosphorylation in sickle cell disease, increased RBC deformability, and enhanced blood flow through microcapillaries [20]. Src tyrosine kinases transactivated by GPCRs might also phosphorylate other RBC proteins, as well. Shear stress-activated GPCRs might induce serine phosphorylation through the classical mechanism of AC/cAMP signaling and tyrosine phosphorylation mediated by Src kinases in the RBC proteome. Intracellular signaling and downstream effects of PKA would be suppressed by inhibiting AC and PKA by SQ22536 and H89 dihydrochloride, respectively. According to our Western blot results, SQ22536 or H89 dihydrochloride implementations did not significantly alter PKA-mediated serine phosphorylation of the cytoskeletal proteins (Figure 5B). However, the same inhibitors increased serine phosphorylation of the RBC proteome compared to the controls (Figure 5D). The elevation in serine phosphorylation by inhibiting cAMP signaling might be due to ‘’crosstalk’’ between different signaling pathways, such as cGMP-dependent mechanisms via PDEs (Figure 6) [65,66,67]. Intracellular cGMP can activate extracellular signal-regulated kinase (ERK), which is a member of MAPK that facilitates serine phosphorylation [68]. Zennadi et al. demonstrated that ERK targets RBC proteins, such as protein 4.1, dematin, and adducins, that might be responsible for cytoskeletal disorganization and contribute to sickle cell pathophysiology [69]. Although we did not find a significant increase in serine phosphorylation of the cytoskeletal proteins, elevated serine phosphorylation in intact cells indicates that ERK might target other cytoplasmic proteins, as well. In addition, tyrosine phosphorylation significantly increased in the cytoskeletal proteins (Figure 5A) and in the total phosphorylation status (Figure 4A) by inhibiting AC and PKA. The possible explanation of the enhanced tyrosine phosphorylation of RBC membrane proteins is the activation of receptor tyrosine kinases (RTK) or non-receptor tyrosine kinases (NRTK) through unknown mechanisms. Although RTK is activated in a ligand-binding way, activation of NRTK, such as Src family kinases, involves a much more complex mode of action, possibly transactivation by GPCRs, enabling transphosphorylation [70].

On the other hand, the inhibition of PDE by pentoxifylline maintained cAMP signaling and increased serine phosphorylation in the intact cells, as expected. However, serine phosphorylation of the cytoskeletal proteins was decreased. Typically, PDE is a negative feedback mechanism for the cAMP/PKA pathway. Another negative regulation was defined where the action of PKA is counterbalanced by specific protein phosphatases, including PP1 and PP2A [71,72]. The inhibition of PDE could lead to the continuous activation of PKA that prompts the activities of the phosphatases, which prevents further phosphorylation of the cytoskeletal proteins. Interestingly, pentoxifylline significantly increased tyrosine phosphorylation of the cytoskeletal proteins and the RBC proteome, as well (Figure 5). Pentoxifylline is a non-selective PDE inhibitor and maintains cAMP or cGMP signals that mostly favor serine phosphorylation. However, studies have shown that the inhibition of PDE by pentoxifylline could also lead to an increase in tyrosine phosphorylation in spermatozoa associated with hyperactivated motility [73,74]. This mechanism was explained in terms of the PKA-stimulated activation of Src tyrosine kinase facilitating tyrosine phosphorylation, since PKA induces Src phosphorylation at serine residues and leads to its activation in multiple cell types [75,76]. A similar mechanism might be responsible for the enhanced tyrosine phosphorylation of the RBC proteome mediated by Src kinase by PDE inhibition (Figure 6).

Several studies have supported the feasibility of the cAMP/PKA pathway as a target for treatment of a variety of diseases, such as cancers [77,78], cardiovascular diseases [79], diabetes [80], and Alzheimer’s disease [81]. Aberrant cAMP signaling through dysregulation of cAMP compartmentalization contributes to cardiac remodeling and hypertrophy [82]. Current studies aim at determining the involvement of the cAMP-binding protein EPAC in the process of cardiac pathological remodeling under chronic β-AR stimulation [83]. The cAMP/PKA pathway also regulates the activities of key molecules involved in insulin secretion, including GLUT2, K-ATP, and Cav channels. PKA-dependent phosphorylation suppresses the catalytic activity of GLUT2 and reduces glucose uptake into β-cells [84], contributing to the pathophysiology of Type 2 Diabetes. In addition, hydroxyurea induces fetal hemoglobin expression by activating the cAMP pathway by cAMP- and cGMP-dependent mechanisms, producing redundancy in the response of HbF to hydroxyurea [85]. When cAMP signaling was activated in adult erythroid cells, fetal hemoglobin was induced at high levels and associated with reduced expression of BCL11A, a silencer of the β-globin gene [86]. These studies suggest novel therapeutic approaches for hemoglobinopathies, such as sickle cell disease and beta-thalassemia, by targeting the cAMP/PKA pathway.

In the present study, we identified changes in the phosphorylation state by cAMP signaling mechanisms in RBCs exposed to physiological shear stress and a possible relationship with shear-induced deformability. We demonstrated that physiological shear stress induces mild phosphorylation of serine and tyrosine residues in the RBC proteome, which improves RBC deformability. On the other hand, the modulation of the cAMP/PKA signaling pathway under shearing conditions leads to enhanced phosphorylation, particularly tyrosine residues of the RBC proteome, which impairs shear-induced deformability. Therefore, shear-induced deformability is regulated by phosphorylation events in the cytoskeletal proteins. Previous studies showed that the phosphorylation of protein 4.1 weakens its association to the cytoskeleton, which could improve RBC deformability [87,88]. However, the phosphorylation of band 3 anchors it to the cytoskeleton, which potentially influences membrane stability and impairs RBC deformability [89,90]. Recently, Moura et al. demonstrated that the inhibition of Lyn and GSK3 kinases decreases RBC deformations during capillary transit [34]. Furthermore, Lyn and GSK3 induce tyrosine and serine phosphorylation, respectively. These phosphorylation events may regulate the resistance of RBCs to shear stress and modulate shear-induced deformability, although they target different residues. Our results are consistent with the findings of Moura et al. that the inhibition of signaling molecules in the cAMP pathway triggered significant phosphorylation and impaired shear-induced deformability. In addition, our results show that the extent of deformability depends on the level of phosphorylation. Although phosphorylation events were previously shown to regulate RBC deformability, the relationship between deformability and phosphorylation levels has not been suggested to date. The improvements of RBC deformability are accompanied by mild phosphorylation of the proteins; as phosphorylation increases, shear-induced deformability is impaired.

In summary, the cAMP/PKA signaling pathway could regulate RBC deformability during capillary transit by triggering significant alterations in the phosphorylation status of RBCs. Furthermore, the phosphorylation level may regulate shear-induced deformability, plausibly on the binding sites of the cytoskeletal proteins via protein kinases in the cAMP/PKA pathway. These results enable us to understand shear-induced deformability through changes in phosphorylation and suggest that targeting cAMP signaling may represent a novel therapeutic approach for circulatory disorders where RBC deformability is substantially impaired.

## Figures and Tables

**Table 1:** Proteins identified by mass spectrometric analysis. Selected proteins are in bold.

UniProt No	Protein	Coverage (%)	UP ^1^	Peptide	PSM ^2^
P60709	Actin, cytoplasmic 1	41.87	9	12	30
P11171	Protein 4.1	28.82	21	24	59
P02549	Spectrin alpha chain, erythrocytic 1	33.11	75	78	179
H0YK48	Tropomyosin alpha-1 chain	65.32	4	24	60
O43396	Thioredoxin-like protein 1	30.10	7	8	24
P28289	Tropomodulin-1	60.72	23	24	71
P17980	26S protease regulatory subunit 6A	35.54	12	15	30
Q14254	Flotillin-2	54.91	21	24	57
P60900	Proteasome subunit alpha type-6	43.50	9	10	25
P62879	Guanine nucleotide-binding protein G(I)/G(S)/G(T) subunit beta-2	37.65	4	11	36
O75955	Flotillin-1	31.15	12	12	26
Q08495	Dematin	62.96	24	29	302
P02549	Spectrin alpha chain, erythrocytic 1	70.28	172	192	732
P17980	26S protease regulatory subunit 6A	53.99	17	21	49
P02549	Spectrin alpha chain, erythrocytic 1	44.11	99	111	307

^1^ Unique peptide. ^2^ Peptide sequence match.

## Data Availability

The data presented in this study are available on request from the corresponding author.

## Supplementary Materials

The following supporting information can be downloaded at: https://www.mdpi.com/article/10.3390/cells11071250/s1. Figure S1: Parameterization of EI–SS curves of different treatments (SQ, H89, and pentoxifylline). SS1/2, EImax, and SS1/2:EImax values are shown in panels A, B, and C, respectively. Data are represented as means ± SD of n = 10, * *p* < 0.05, Figure S2: Expression levels of RBC membrane proteins by different treatments. CB, CA, SQ, H, and P refer to Control before SS (blue bars), Control after SS (red bars), SQ22536 (green bars), H89 (purple bars), and Pentoxifylline (orange bars), respectively. Panels A, B, C, D, E, and F show dematin, tropomodulin, tropomyosin, spectrin, protein 4.1 and actin, respectively. AU: Arbitrary unit. Data are represented as means ± SD of n = 10, Figure S3: Phosphorylation status in intact RBCs with different drug treatments before 5 Pa SS exposure, Supplementary Table S1: Demographics and hematological parameters of study participants (means ± SD).
