# Supplementary material for: Proteomic Analysis of the Role of the Adenylyl Cyclase–cAMP Pathway in Red Blood Cell Mechanical Responses
Source: Cells. 2022 Apr 6;11(7):1250. doi: 10.3390/cells11071250 (PMC8997765; doi:10.3390/cells11071250)
Supplement: Supplementary file 1 [file cells-11-01250-s001.zip › Supplementary Table S1.pdf]

Supplementary Table S1. Demographics and hematological parameters of study participants (Mean±SD).

Gender (f/m)	5/7
Age	29.67±7.89
White blood cell count (10^3/mm^3)	6.8±1.26
Red blood cell count (10^6/mm^3)	4.7±0.41
Hemoglobin (g/dL)	14.0±1.14
Hematocrit (%)	43.9±3.42
Mean corpuscular volume (μm^3)	93.1±3.06
Mean corpuscular hemoglobin (pg)	29.7±1.05
Mean corpuscular hemoglobin concentration (g/dL)	31.8±0.31
