# Supplementary figures and images for: Proteomic Analysis of the Role of the Adenylyl Cyclase–cAMP Pathway in Red Blood Cell Mechanical Responses
Source: Cells. 2022 Apr 6;11(7):1250. doi: 10.3390/cells11071250 (PMC8997765; doi:10.3390/cells11071250)

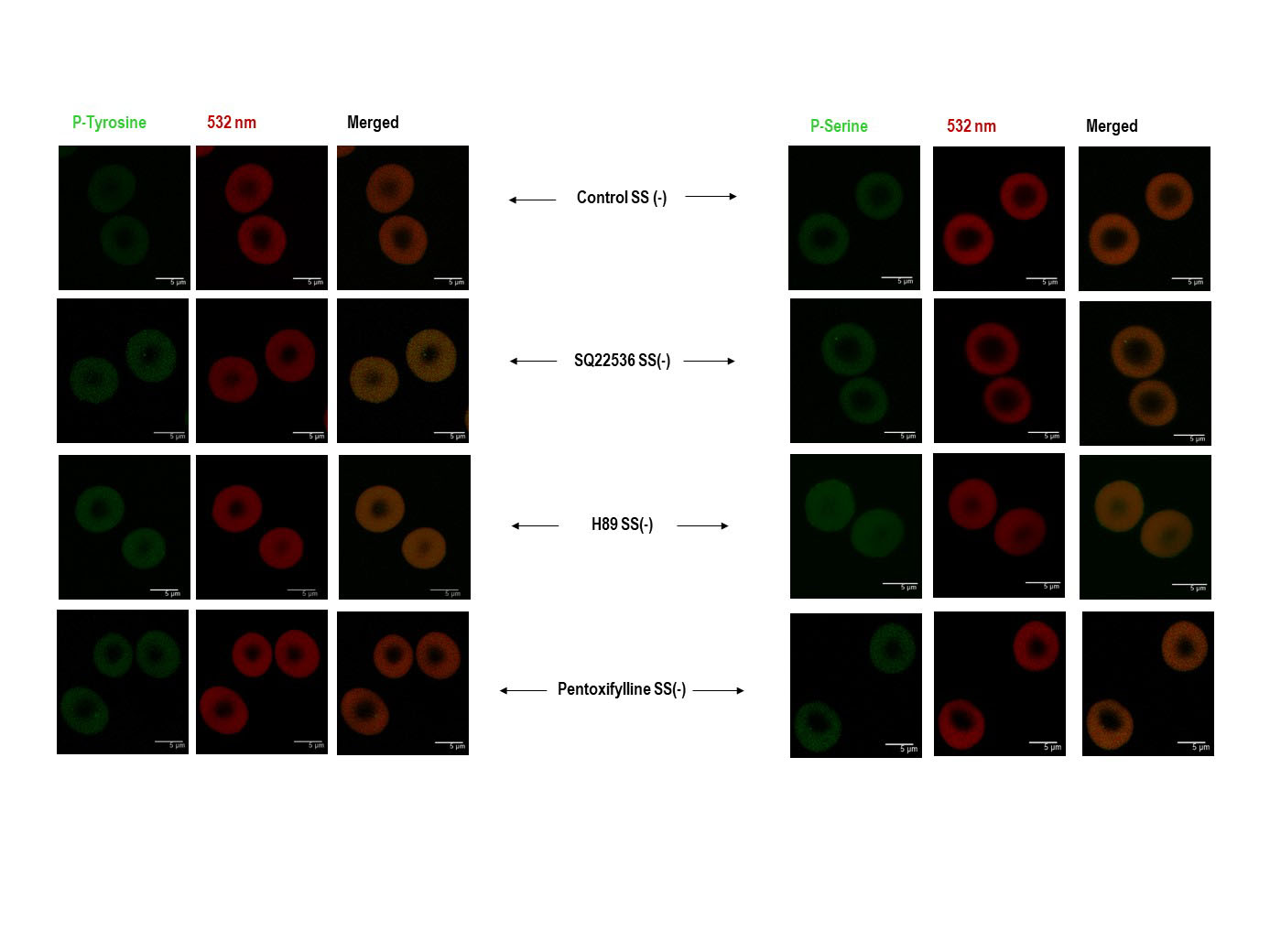

Supplement: Supplementary file 1 [file cells-11-01250-s001.zip › Supplementary Figure S3.jpg]
